# A systematic and meta-analysis review on the diagnostic accuracy of antibodies in the serological diagnosis of COVID-19

**DOI:** 10.1186/s13643-021-01689-3

**Published:** 2021-05-26

**Authors:** Arthur Vengesai, Herald Midzi, Maritha Kasambala, Hamlet Mutandadzi, Tariro L. Mduluza-Jokonya, Simbarashe Rusakaniko, Francisca Mutapi, Thajasvarie Naicker, Takafira Mduluza

**Affiliations:** 1 Department of Biochemistry, University of Zimbabwe, P.O. Box MP 167, Mt Pleasant, Harare, Zimbabwe; 2 Optics and Imaging, Doris Duke Medical Research Institute, College of Health Sciences, University of KwaZulu-Natal, Durban, KwaZulu-Natal South Africa; 3 College of Health Sciences, University of Zimbabwe, Box A178 Mazowe Street Avondale, Harare, Zimbabwe; 4 Institute for Immunology and Infection Research and Centre for Immunity, Infection and Evolution, School of Biological Sciences, Ashworth Laboratories, University of Edinburgh, King’s Buildings, Charlotte Auerbach Rd, Edinburgh, EH9 3JT UK

**Keywords:** Serology, COVID-19, SARS-CoV2, rRT-PCR, IgG, IgM, Specificity, Sensitivity

## Abstract

**Background:**

Serological testing based on different antibody types are an alternative method being used to diagnose SARS-CoV-2 and has the potential of having higher diagnostic accuracy compared to the current gold standard rRT-PCR. Therefore, the objective of this review was to evaluate the diagnostic accuracy of IgG and IgM based point-of-care (POC) lateral flow immunoassay (LFIA), chemiluminescence enzyme immunoassay (CLIA), fluorescence enzyme-linked immunoassay (FIA) and ELISA systems that detect SARS-CoV-2 antigens.

**Method:**

A systematic literature search was carried out in PubMed, Medline complete and MedRxiv. Studies evaluating the diagnostic accuracy of serological assays for SARS-CoV-2 were eligible. Study selection and data-extraction were performed by two authors independently. QUADAS-2 checklist tool was used to assess the quality of the studies. The bivariate model and the hierarchical summary receiver operating characteristic curve model were performed to evaluate the diagnostic accuracy of the serological tests. Subgroup meta-analysis was performed to explore the heterogeneity.

**Results:**

The pooled sensitivity for IgG (*n* = 17), IgM (*n* = 16) and IgG-IgM (*n* = 24) based LFIA tests were 0.5856, 0.4637 and 0.6886, respectively compared to rRT-PCR method. The pooled sensitivity for IgG (*n* = 9) and IgM (*n* = 10) based CLIA tests were 0.9311 and 0.8516, respectively compared to rRT-PCR. The pooled sensitivity the IgG (*n* = 10), IgM (*n* = 11) and IgG-IgM (*n* = 5) based ELISA tests were 0.8292, 0.8388 and 0.8531 respectively compared to rRT-PCR. All tests displayed high specificities ranging from 0.9693 to 0.9991. Amongst the evaluated tests, IgG based CLIA expressed the highest sensitivity signifying its accurate detection of the largest proportion of infections identified by rRT-PCR. ELISA and CLIA tests performed better in terms of sensitivity compared to LFIA. IgG based tests performed better compared to IgM except for the ELISA.

**Conclusions:**

We report that IgG-IgM based ELISA tests have the best overall diagnostic test accuracy. Moreover, irrespective of the method, a combined IgG/IgM test seems to be a better choice in terms of sensitivity than measuring either antibody type independently. Given the poor performances of the current LFIA devices, there is a need for more research on the development of highly sensitivity and specific POC LFIA that are adequate for most individual patient applications and attractive for large sero-prevalence studies.

**Systematic review registration:**

PROSPERO CRD42020179112

**Supplementary Information:**

The online version contains supplementary material available at 10.1186/s13643-021-01689-3.

## Introduction

Coronavirus disease 2019 (COVID-19) is a major contagious pandemic of respiratory disease caused by the severe acute respiratory syndrome coronavirus 2 (SARS-CoV-2), which is also known as novel (new) coronavirus 2019-nCoV [1–3]. The first COVID-19 cases were identified in December 2019 from Wuhan, Hubei Province, China [4]. On November 18 2020, according to the European Centre for Disease Prevention and Control, COVID-19 Situation update, there were 55,743,951 confirmed cases and 1,339,436 deaths reported worldwide [5]. Although the COVID-19 clinical features are not yet fully known and understood, clinicians have reported clinical manifestations that range from asymptomatic cases to patients with mild and severe respiratory illness, with or without pneumonia, fever, cough and shortness of breath. Older people (>65 years) and people of all ages with severe chronic medical conditions such as lung disease, heart disease and diabetes seem to have a higher risk of succumbing to severe COVID-19 illness [6].

Early and accurate diagnostic testing for COVID-19 is critical for tracking the SARS-CoV-2, understanding the virus epidemiology, informing case management, suppressing transmission and for quarantine purposes [7, 8]. The standard diagnostic confirmatory test for COVID-19 is based on the detection of nucleic acids of SARS-CoV-2 by nucleic acid amplification tests, such as real-time reverse-transcriptase polymerase chain reaction (rRT-PCR). The test identifies viral nucleic acids when present in sufficient quantity in sputum, throat swabs and secretions of the lower respiratory tract. In some patients, SARS-CoV-2 RNA detection in blood and oral fluid specimens has been reported; however, limited data is available on adequacy of SARS-CoV-2 detection in these specimens [9]. The rRT-PCR test is time consuming as it takes between 4 to 6 h for completion. It requires expensive specialist equipment, skilled laboratory personal for sample preparation and testing and PCR reagents, creating diagnostic delays and limiting use in real-life situations when rapid diagnosis is required for fast intervention decisions. Therefore, less expensive and easy implementable tests are required for SARS-CoV-2 detection. Another limitation of using rRT-PCR involves the use of swabs from the upper respiratory tract which can be falsely diagnosed as negative due to the poor quality of the sample or acquiring the sample at an incorrect timeframe; notably, viral load in upper respiratory tract secretions peak in the first week of symptoms but may decline below the limit of detection in patients presenting late with symptoms [8, 10–12]. Missing the time-window of viral replication may also provide false negative results. Moreover, after a variable period of time, one expects the rRT-PCR result to become negative due to cessation of viral shedding [13].

False-negative rRT-PCR results are common during diagnosis of SARS-CoV-2. The Fever Clinic of the Beijing Haidian Hospital collected data from January 2020 which indicated that only two out of ten negative cases diagnosed by rRT-PCR test were confirmed to be true positive for COVID-19. This yielded an approximately 20% false-negative rate of rRT-PCR [12]. Zhang et al. also showed that the current strategy for the detection of viral RNA in oral swabs used for SARS-CoV-2 diagnosis is not 100% accurate. The presence of the virus has been detected in anal swabs or blood samples of patients whilst their oral swabs diagnosis reports a negative result. This observation implies that a patient cannot be discharged based purely on oral swab samples being negative [14].

A false negative diagnosis may have grave consequences, especially at this stage of the COVID-19 pandemic by allowing SARS-CoV-2 infected patients to spread the infection thereby hampering the efforts to contain the spread of the virus [8]. Additional screening methods that can detect the presence of infection despite lower viral titres are highly beneficial to ensure timely diagnosis of all COVID-19 patients. Detection of serum specific anti-SARS-CoV-2 antibodies, both immunoglobulin G (IgG) and M (IgM), which are produced rapidly after the infection provides an alternative highly sensitive and accurate solution and compensates for the limitations of rRT-PCR. The serological methods could also be a more practical alternative to chest CT [8, 15, 16]. Immunoglobulin G antibodies permit the use of serological tools to better understand the overall rate of COVID-19 infections including the rate of asymptomatic infections [8].

However, the dynamics of blood or serum antibodies in the cases of COVID-19 are not well evaluated. Currently, the serological dynamics of COVID-19 patients remain limited. Also, before diagnostic assays are widely deployed, their performance characteristics need to be evaluated. Therefore, the objective of this review was to evaluate the diagnostic accuracy of IgG and IgM (together or separately) based point-of-care (POC) lateral flow immunoassay (LFIA), chemiluminescence enzyme immunoassay (CLIA), fluorescence enzyme-linked immunoassay (FIA) and enzyme-linked immunosorbent assay (ELISA) to detect antigens against SARS-CoV-2.

## Review questions

The primary research question of this systematic review was ‘What is the diagnostic accuracy of antibody serology tests for COVID-19 using the bivariate model and the hierarchical summary receiver operating characteristic curve (HSROC) model?’

## Materials and methods

We conducted a systematic review and meta-analysis in accordance with the recommendations of the Preferred Reporting Items for Systematic Reviews and Meta-Analyses of Diagnostic test accuracy (PRISMA-DTA) [17] (Additional file 1). We used the Cochrane recommendations to report systematic reviews and meta-analyses of studies on diagnostic accuracy [18]. We also used protocols from published systematic and meta-analysis reviews to develop our protocol [19–22]. The developed systematic review protocol was registered in the International Prospective Register of Systematic Reviews registration number CRD42020179112.

### Eligibility criteria

Cross-sectional studies would be the ideal study type to answer our review questions. However, as we anticipated that serological diagnosis cross-sectional studies for COVID-19 would be very sparse, we decided to include case-control studies. The inclusion criteria comprised studies in which the study population (*n*≥10) were subjected to COVID-19 rRT-PCR testing or genetic sequencing as the reference standards and either one or all of the following serological tests; point-of-care (POC), lateral flow immunoassay (LFIA), chemiluminescence enzyme immunoassay (CLIA), fluorescence immunoassays (FIA) and enzyme-linked immunoassay (ELISA). Studies that used chest CT images, epidemiological history, well-defined clinical features accompanied by rRT-PCR as a reference standard were included. The diagnostic accuracy of the tests was defined as the primary outcome. Original studies were included without restriction based on language or geographical location. We included studies between 1 January 2020, and 27 April 2020. Animal studies, in vitro*-*based studies and survey studies investigating seroprevalence were excluded from the study.

### Information sources and search strategies

The following databases were searched for studies: MEDLINE Complete (EBSCO), PubMed, and MedRxiv (a preprint server for health sciences which distributes complete but unpublished manuscripts). We performed the search strategy on studies dated until 29 April 2020. The data bases were searched using predefined keywords: COVID-19 and serologic test and their synonyms. Appendix 1 illustrates the search strategy for PubMed, which was adapted for the other data bases. Additional studies were identified by contacting experts in the field and by searching reference lists from primary studies, review articles and textbook chapters.

### Study selection and data extraction

Two authors (AV and HM) assessed the titles identified by the search, excluding those obviously irrelevant to the serological diagnosis of COVID-19. Letters, review articles and articles clearly irrelevant based on examination of the abstract and other notes and duplicates were excluded next. The eligibility of the remaining potentially relevant articles was judged on full-text publications**.**

Data extraction was conducted independently by two authors (AV and HM) to avoid bias and discrepancies and was resolved by discussion. Where an agreement could not be reached, a third author was consulted. Where it remained unclear whether a study is eligible for inclusion, it was then excluded. Whilst extracting data, authors also had to decide whether a study was a case-control or a cross-sectional study. The following data were extracted.

- Study authors and publication year
- Study design
- Case definition (inclusion/exclusion criteria)
- Participant demographics
- Reference standard (including criteria for positive test)
- Index tests [cutoff values (prespecified or not) and whether the test was a commercial of in-house test]
- Geographical location of data collection
- Index/reference time interval
- Distribution of severity of disease in those with target condition
- Other diagnoses in those without target condition
- Numbers of TPs, FNs, FPs and FNs

### Other considerations and exclusion criteria

Studies, from which a 2×2 table containing true positives, false positives, false negatives and true negatives could not be drawn, were excluded. Furthermore, studies that were too unspecific in their reporting to ensure that they fulfilled the above criteria, were excluded.

### Assessment of methodological quality

The QUADAS-2 tool was used to assess the methodological quality of all studies included in this systematic review [23]. QUADAS-2 consists of four key domains: patient selection, index test, reference standard and flow and timing. We assessed all domains for risk of bias (ROB) potential and the first three domains for applicability concerns. Risk of bias was judged as ‘low,’ ‘high’ or ‘unclear’. Details are shown in Appendix 2. Two review authors (AV and HM) independently completed QUADAS-2. The divergences were resolved by consensus amongst the researchers.

### Statistical analysis and data synthesis

#### Diagnostic accuracy

For each study, we constructed 2 × 2 tables for true positives (TP), true negatives (TN), false positives (FP) and false negatives (FN). Where only sensitivity and specificity estimates were reported, we derived the two-by-two table from the reported data. We constructed forest plots displaying sensitivity and specificity of the index tests from contingency tables assuming that the reference method was 100% sensitive and specific. Data were entered into the Review Manager (RevMan) software for Windows v.5.3 (Cochrane Collaboration, Copenhagen, Denmark) and forest plots were created with 95% confidence interval (CI) for sensitivity and specificity for each study.

Studies were submitted to meta-analysis when three conditions were met: sample size was greater than 20; sensitivity and specificity were available for the index and control group was included in the analysis. We used the two recommended random-effects hierarchical methods: the bivariate model and the hierarchical summary receiver operating characteristic (HSROC) model for performing the meta-analysis. The focus of the bivariate model is estimation of a summary point (summary sensitivity and specificity). HSROC model is on estimating an SROC curve [24]. The summary estimates of sensitivity and specificity and 95% CI and the HSROC were calculated using OpenMeta-Analyst for windows 10 (open-source, cross-platform for advanced meta-analysis).

#### Investigations of heterogeneity

We investigated heterogeneity by adding antigen type as the covariate. The following approach was taken: Firstly, the variation in accuracy between IgG or IgM or IgG-IgM based LFIA, CLIA and ELISA serological testing was analysed (Table 2). Then, the effect of the antigen type was investigated using subgroup meta-analysis in OpenMeta-analyst. *I*^^2^ values close to 0% were considered as having no heterogeneity between studies; values close to 25 %, low heterogeneity; values close to 50%, moderate heterogeneity and values close to 75%, high heterogeneity between studies [25].

#### Assessment of publication bias

In this review, we did not assess for reporting bias. The studies included in our meta-analysis showed a lot of heterogeneity; therefore, assessments for reporting bias may not yield conclusive results. This was adopted from the approach used by Ochodo et al. [26].

#### Test sensitivity by time since onset of symptoms

We stratified data by days since COVID-19 symptom onset to specimen collection. Then, we constructed forest plots (95 % CI) displaying test sensitivity by time since onset of symptoms using the RevMan software for Windows v.5.3 (Cochrane Collaboration, Copenhagen, Denmark).

## Results

### Study inclusion

**Fig. 1:**
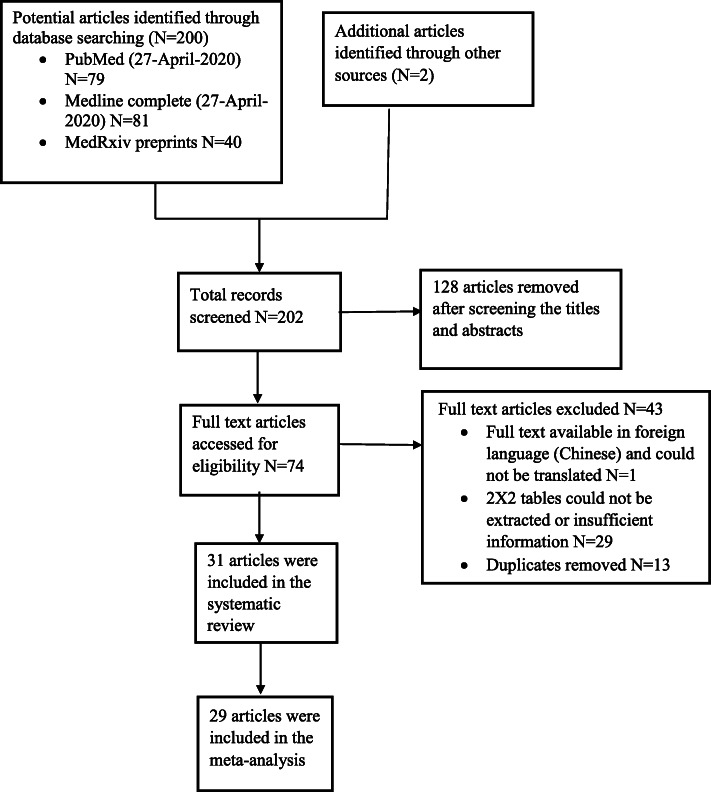
PRISMA flow diagram for selection of articles for meta-analysis

The results of the search and selection process are presented in Fig. 1. A total of 202 articles were identified. Amongst these, 40 were MedRxiv preprints and 162 were fully published articles from MEDLINE Complete (EBSCO) and PubMed. Two articles were identified from other sources for example manual search. After abstract/title exclusion and removing duplicates, 74 articles were submitted to full-text screening and 31 of these were included for the systematic review. Most articles were excluded because they did not present sufficient data hence it was not possible to extract data to construct 2 × 2 table and 1 article was excluded because it was only available in Chinese. A total of 29 articles describing the results of 99 independent studies/data sets (19, 23 and 57 investigating LFIA, CLIA and ELISA respectively) were eligible for the meta-analysis.

### Characteristics of the studies

The general characteristics of the included articles are presented in Table 1. All the published articles (*n* = 14) included in the review were published in 2020 because COVID-19 is an emerging disease. The 17 unpublished articles were MedRxiv preprints which have been submitted to different journals for publication. Twenty five articles included in the review had a case-control design, comparing a group of well-defined cases with a group of healthy controls or controls with diseases or COVID-19 rRT-PCR negative patients, and only six studies were cross sectional studies. One study had no control group and was excluded in the meta-analysis [27]. Most of the studies (*n* = 22) were conducted in China where the COVID-19 pandemic began and 3 studies were conducted in Italy whilst, USA whilst UK, Denmark, Germany, Spain and Japan each conducted one study.

**Table 1:** The general characteristics of the studies included in the review

Study ID	Country	Antibody type	Antigen type	Commercial	Reference standard	Index test	Control group/comparison group
Kai-Wang To [27] ^P, CS^	China	IgG and IgM	S and N	Inhouse	rRT-PCR	ELISA	No control
Cassaniti [28] ^P, CS^	Italy	IgG, IgM and IgG-IgM	S	Commercial	rRT-PCR	POC LFIA	Patients with fever and respiratory syndrome/RT-PCR negative
Duchuan Lin [29]	China	IgG, IgM and IgG-IgM	N	Inhouse	Epidemiological risk/clinical features/rRT-PCR	CLIA	Healthy individuals and tuberculosis patients
Jie Xiang [30]	China	IgG, IgM and IgG-IgM		Commercial	rRT-PCR	ELISA and POC LFIA	Healthy individuals
Li Guo [13] ^P^	China	IgM	N	Inhouse	Deep sequencing and rRT-PCR	ELISA	Adult patients with acute lower respiratory tract infections (ALRTIs)
Rui Liu [31] ^CS^	China	IgM	N	Inhouse	rRT-PCR		COVID-19 rRT-PCR negative patients
Wanbing Liu [32] ^P^	China	IgG, IgM and IgG-IgM	S and N	Commercial	rRT-PCR	ELISA	Healthy individuals
Xuefei Cai [33]	China	IgG, IgM and IgG-IgM	S	Inhouse	rRT-PCR	Peptide-based magnetic CLIA	Mixed diseases and healthy controls
Yu bao Pan [34] ^CS^	China	IgG, IgM and IgG-IgM		Commercial	rRT-PCR	POC LFIA	COVID-19 rRT-PCR negative patients
Yujiao Jin [35] ^P^	China	IgG, IgM and IgG-IgM	S-N	Commercial	rRT-PCR	CLIA	Patients with suspected SARS-CoV-2 infection but with negative rRT-PCR results
Zhao [36] ^P^	China	IgG and IgM	S	Commercial	Chest CT images/epidemiological history/clinical diagnosis/rRT-PCR	ELISA	Healthy individuals
Zhengtu Li [37] ^P^	China	IgG, IgM and IgG-IgM	S	Commercial	rRT-PCR	POC LFIA	Healthy individuals
Rongqing Zhao [38]	China	IgG-IgM	S	Inhouse	Not clear but all cases were confirmed COVID-19 patients	ELISA	Healthy individuals (samples collected before and during the COVID-19 pandemic)
Pingping Zhang [39]	China	IgG-IgM	S	Inhouse	rRT-PCR	POC LFIA	COVID-19 rRT-PCR negative patients
Paradiso [40]	Italy	IgG-IgM	S	Commercial	rRT-PCR	POC LFIA	Patients with COVID-19 disease orienting-symptoms but rRT-PCR negative
Huan Ma [41]	China	IgA, IgG, IgM, IgG-IgM and Ab	S and N	Inhouse	rRT-PCR	CLIA	Healthy individuals, COVID-19 suspected individuals and mixed disease group
Qian [15]	China	IgG and IgM	S-N	Commercial	rRT-PCR	CLIA	Healthy individuals and hospitalised individuals
Ling Zhong [42] ^P^	China	IgG and IgM		Inhouse	rRT-PCR	CLIA and ELISA	Healthy individuals
Jiajia Xie [43] ^P, CS^	China	IgG and IgM	E-N	Commercial	Chest CT images/epidemiological history/clinical diagnosis/rRT-PCR	CLIA	Clinically confirmed COVID-19 rRT-PCR negative patients
Infantino [44] ^P^	Italy	IgG and IgM	S-N	Commercial	rRT-PCR	CLIA	Mixed diseases patients and blood donors pre-COVID-19
Adams [45]	UK	IgG, IgM and IgG-IgM	S	Inhouse (ELISA) and Commercial (LFIA)	rRT-PCR	ELISA and POC LFIA	Healthy blood and ICU cerebral organ donors before the COVID-19 pandemic
Lassaunière [46]	Denmark	IgA, IgG and Ab	S	Commercial	rRT-PCR	ELISA and POC LFIA	Healthy individuals and mixed diseases patients (including acute respiratory tract infections caused by other corona viruses and non-corona viruses
Qiang Wang [47] ^P^	China	IgG, IgM and IgG-IgM		Commercial	Chest CT images/epidemiological history/clinical diagnosis/rRT-PCR	ELISA and POC LFIA	COVID-19 clinical negative mixed diseases patients
Fei Xiang [30] ^P^	China	IgG and IgM	N	Commercial	rRT-PCR	ELISA	Healthy blood donors or from patients with other disease hospitalised
Bin Lou [48]	China	IgG, IgM and Ab	S and N	Commercial	rRT-PCR	ELISA, CLIA and POC LFIA	Healthy Individuals
Lei Liu [49]	China	IgG-IgM	N	Commercial	rRT-PCR	ELISA	Randomly selected ordinary patients and healthy blood donors
Imai [50]	Japan	IgG, IgM and IgG-IgM		Commercial	rRT-PCR	POC LFIA	Non-COVID-19 patients (from April to October 2019
Pérez-García [51]	Spain	IgG, IgM and IgG-IgM		Commercial	rRT-PCR	POC LFIA	Healthy individuals (samples collected before the COVID-19 pandemic)
Zhenhua Chen [52] ^P^	China	IgG	N	Inhouse	rRT-PCR	POC LFIA	Clinically suspicious for the presence of anti-SARS-CoV-2
Dohla [53] ^P, CS^	German	IgG, IgM and IgG-IgM		Commercial	RT-qPCR	POC LFIA	COVID-19 RT-qPCR negative patients
Burbelo [54]	USA	Ab	S and N	Inhouse	RT-PCR	LIPS	Subjects with COVID-19-like symptoms or household contacts of persons with COVID-19 (not tested by PCR), and blood donors who donated samples before 2018.

Studies with P superscripts were published articles and without P superscripts were MedRxiv preprints

Studies with CS superscripts are cross sectional studies and without CS superscripts are case control studies

IgG-IgM means that either one of them or both were detected in serum

Ab means total antibodies

Most articles (*n* = 26) included in the review clearly stated that the gold standard nucleic acid tests (rRT-PCR or deep sequencing) were used as the reference standard. However, five articles used a combination of epidemiological risk, clinical features, chest CT images and rRT-PCR. In one article, the reference standard used was not stated but all the patients in the study were COVID-19 patients [38].

Point-of-care (POC) lateral flow immunoassays (LFIA) were used in 14 articles, CLIA were used in 9 articles and ELISA were used in 13 articles. We did not identify articles using FIA that met our inclusion criteria. One study did not specify the serological assay used and it was excluded from the review [31]. One study used a LIPS which is performed in solution, thus maintaining the native antigen conformation [54]. Most of the serological assay test kits were commercial (*n* = 21) and 12 were in-house. Three SARS-CoV-2 antigens, Spike protein (S), nucleocapsid protein (N) and envelope protein (E) were used together or separately in studies included in the review. The spike protein and nucleocapsid were used as the antigen in 9 articles and 6 articles respectively. Five articles used both S and N as the antigens separately. In 3 articles, S and N antigens (S-N) were used together as the antigen. In 1 article, N and E antigens (N-E) were used together as the antigen. In 7 articles, the name of antigen used was not given.

### Methodological quality of included studies

**Fig. 2:**
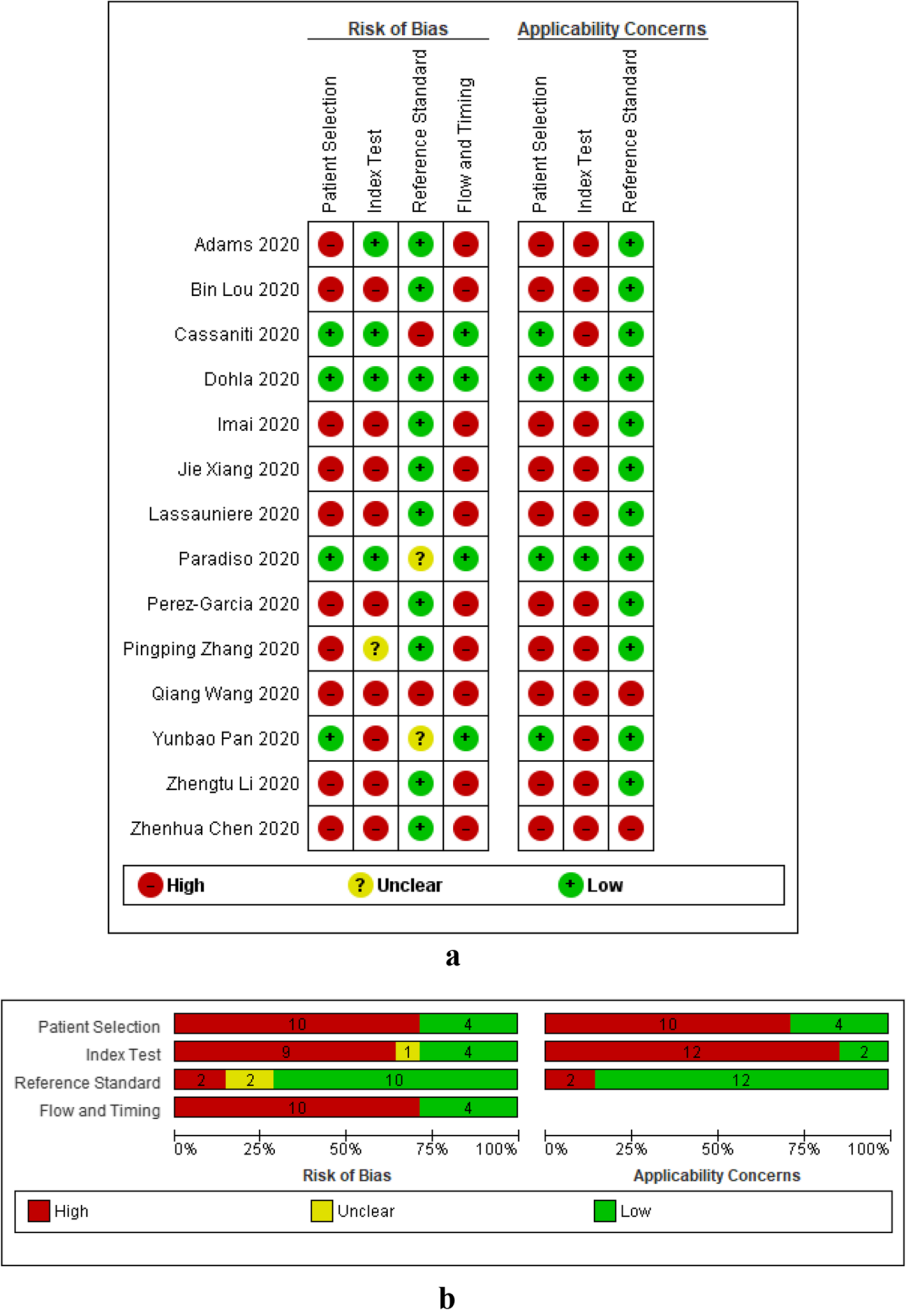
LFIA methodological quality summary table and graph. **a** Risk of bias and applicability concerns summary: review authors’ judgements about each domain for each included study. **b** QUADAS-2 bias assessment and QUADAS-2 applicability assessment item presented as percentages across all included studies. On the left, risk of bias graph and on the right applicability concerns graph. **c** Risk of bias and applicability concerns summary: review authors. Low, low risk of bias; high, high risk of bias; unclear, bias is unclear

**Fig. 3:**
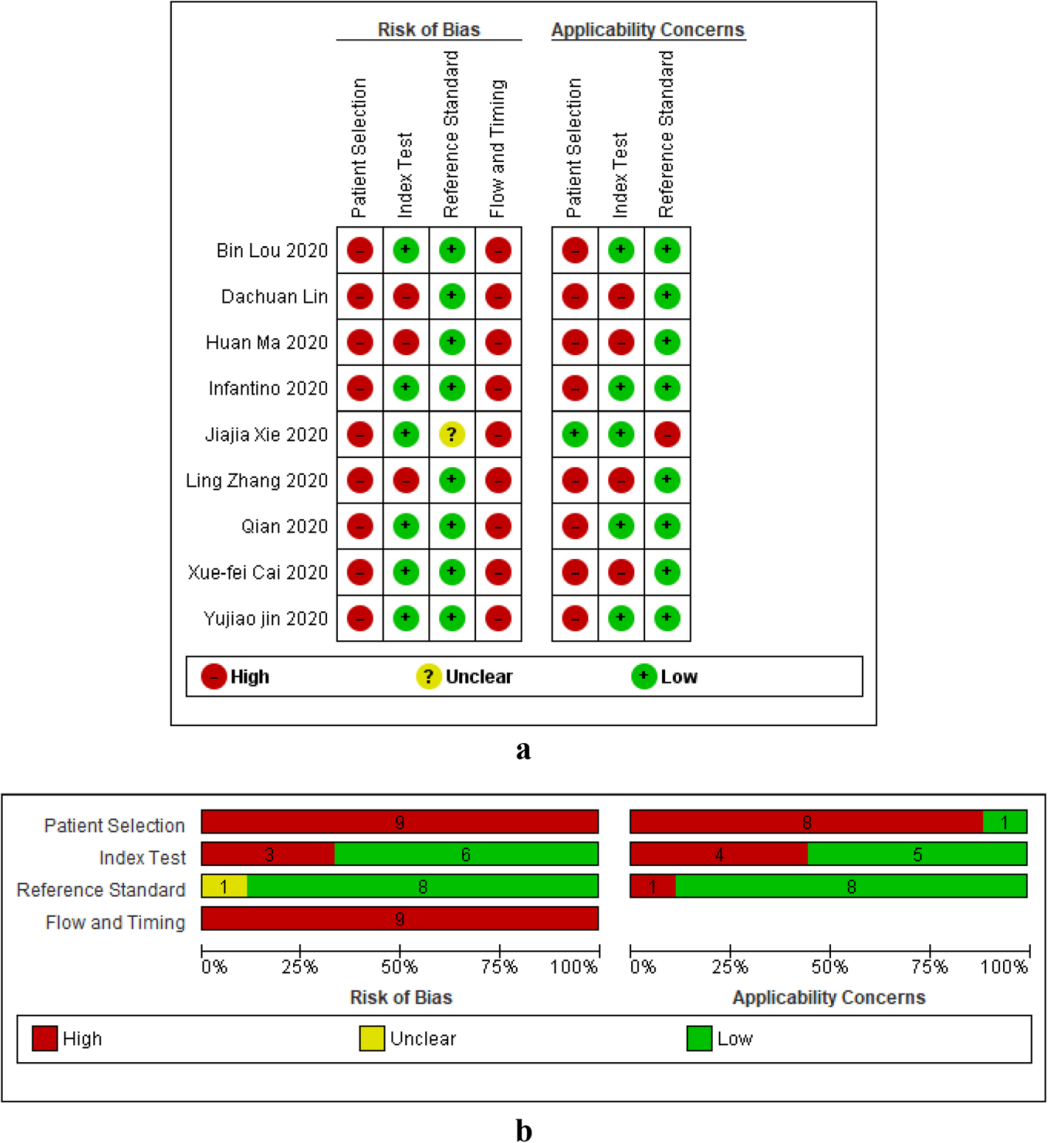
CLIA methodological quality summary table and graph. **a** Risk of bias and applicability concerns summary: review authors’ judgements about each domain for each included study. **b** QUADAS-2 bias assessment and QUADAS-2 applicability assessment item presented as percentages across all included studies. On the left, risk of bias graph and on the right applicability concerns graph. **c** Risk of bias and applicability concerns summary: review authors. Low, low risk of bias; high, high risk of bias; unclear, bias is unclear

**Fig. 4:**
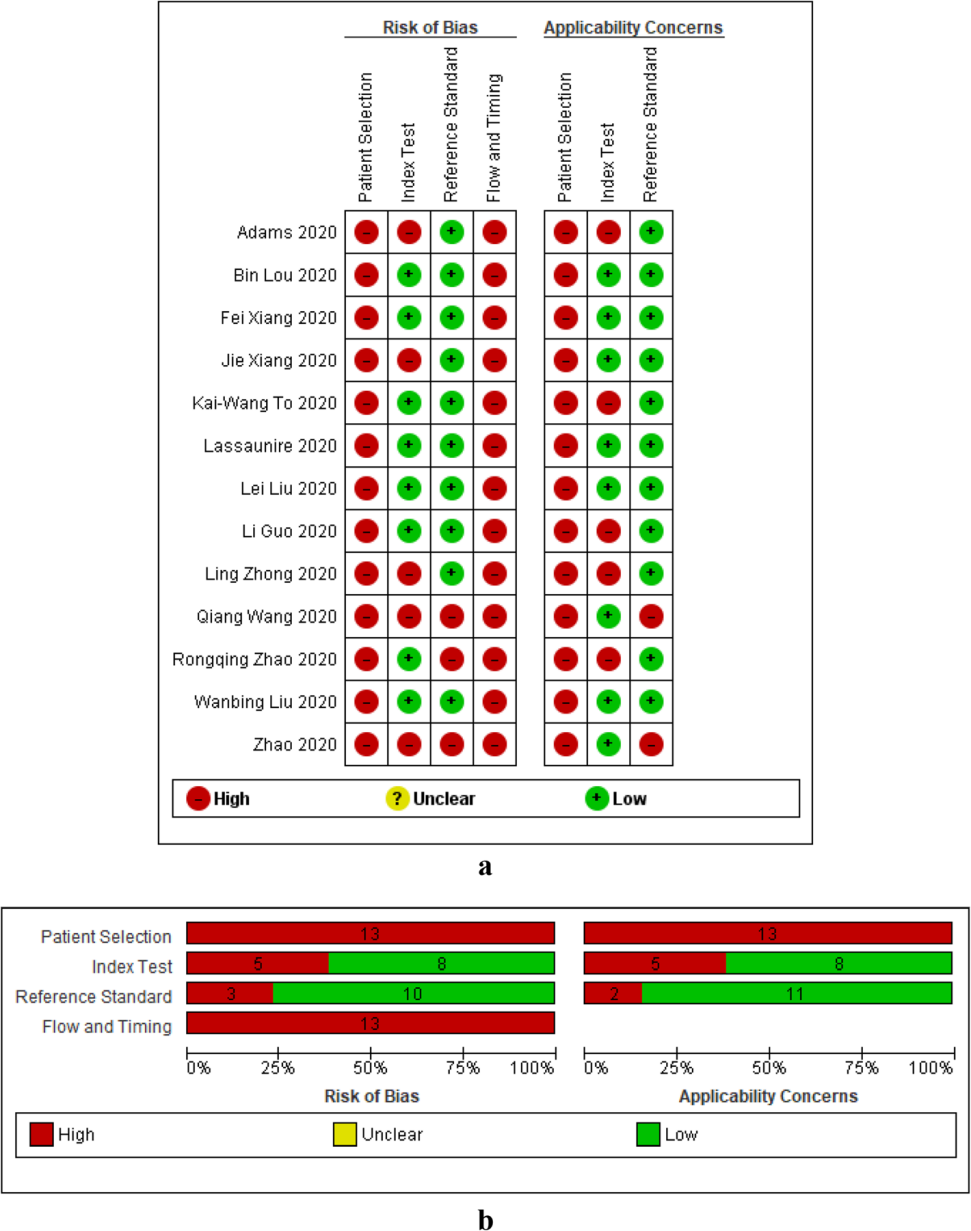
ELISA methodological quality summary table and graph. a Risk of bias and applicability concerns summary: review authors’ judgements about each domain for each included study. **b** QUADAS-2 bias assessment and QUADAS-2 applicability assessment item presented as percentages across all included studies. On the left, risk of bias graph and on the right applicability concerns graph. **c** Risk of bias and applicability concerns summary: review authors. Low, low risk of bias; high, high risk of bias; unclear, bias is unclear

The methodological quality of the included studies for the IgG or IgM or IgG-IgM based LFIA, CLIA and ELISA summarised across all studies are shown in Figs. 2b, 3b and 4b. Figures 2a, 3a and 4a show for the risk of bias and applicability concerns summary results for the LFIA, CLIA and ELISA individual studies respectively. None of the studies included in this review had low risk of bias in all four QUADAS-2 domains. Generally, case control studies were of high risk of bias and high concern in the patients and timing and flow domains and cross sectional studies were of low risk of bias and low concern in all domains.

### Patient selection domain

Generally, most studies included were at risk of bias and had high concerns regarding applicability. Studies were mostly case control studies and they did not include a consecutive or random series of participants implying that the patients that were included are not representative for clinical use. All thirteen ELISA studies were at high risk of bias and had high concerns regarding applicability. For CLIA, all the 9 studies included had high risk of bias and only 1 cross sectional study had low applicability concerns. Generally, LFIA had more studies (*n* = 4) with low risk of bias and applicability concerns in the patient selection domain because there were 4 LFIA cross-sectional studies.

### Index test domain

The LFIA studies had a high risk of bias (9/14) and high applicability concerns (12/14) in the index test domain. The high risk of bias was due to no blinding between the index test and the reference test. The high applicability concerns were due to tests using serum or plasma instead of whole blood which would make the test less amenable to use at the point of care. The CLIA and ELISA studies generally had a low risk of bias (6/9 and 8/13 respectively). This was because most studies were automated and had a pre-specified threshold (cut-off value to decide whether a test is positive or negative). The studies that had high risk of bias did not have a pre-specified threshold. Likewise, CLIA and ELISA studies generally had low applicability concerns in the index test domain (5/9 and 8/13 respectively) because they used commercial index tests.

### Reference standard domain

Like the index test domain, studies generally had a low risk of bias (10/14, 8/9 and 10/13 for LFIA, CLIA and ELISA respectively) in the reference standard domain. Generally, the studies were of low applicability concern, 10/14, 8/9 and 11/13 for LFIA, CLIA and ELISA respectively.

### Flow and timing domain

All the CLIA (*n* = 9) and ELISA (*n* = 13) studies were at high risk of bias in the flow and timing domain. These studies were all case control studies. Most of the LFIA studies were also at a high risk of bias; however, 4 cross sectional LFIA studies were at low risk of bias.

### Quantitative synthesis and meta-analysis

**Fig. 5:**
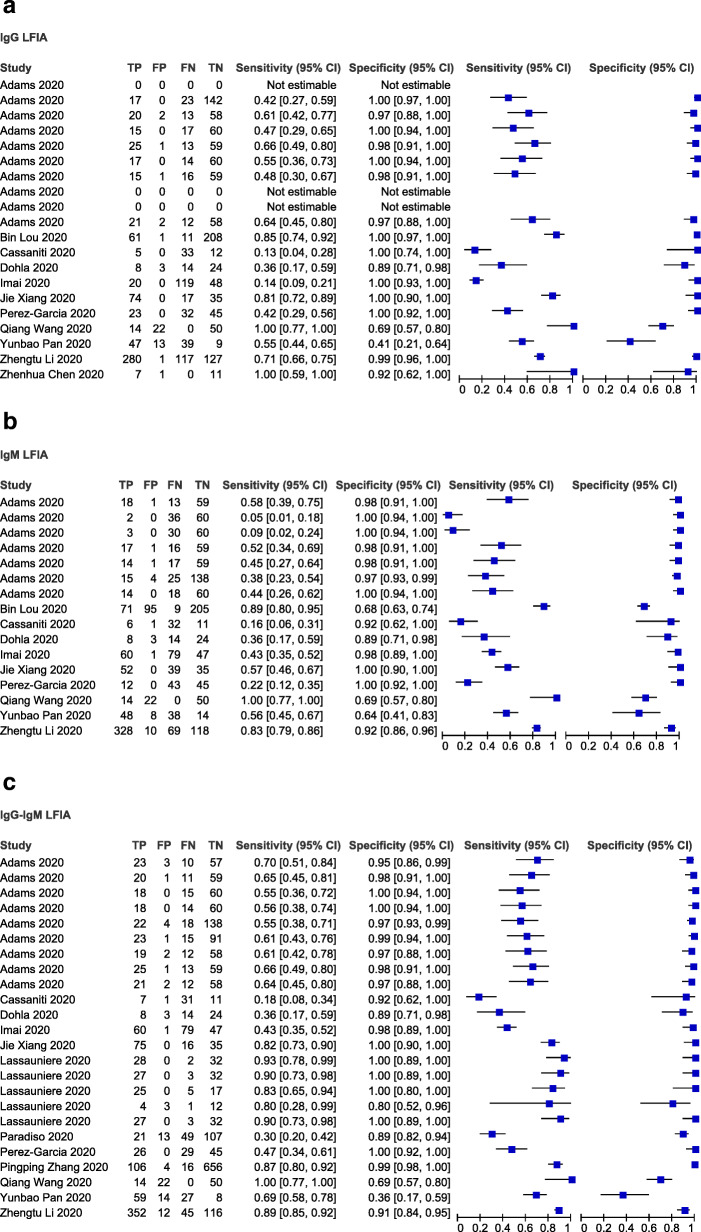
Forest plot of sensitivity, specificity and heterogeneity of serological LFIA diagnosis of COVID-19. **a** Forest plot for the IgG LFIA. **b** Forest plot for the IgM based LFIA. **c** Forest plot for the IgG-IgM based LFIA

Firstly, we considered performance of the LFIA devices using rRT-PCR-confirmed cases as the reference standard. The forest plots in Fig. 5 show the sensitivity, specificity range and heterogeneity for the three IgG or IgM or IgG-IgM based LFIA detecting COVID-19 across the included studies. Overall, the sensitivity varied widely across studies in contrast to the specificity which did not vary much except for 2 studies, Yunbao Pan, 2020, and Qiang Wang, 2020, which had the lowest and second lowest specificities respectively. Amongst the IgG based LFIA tests (*n* = 17), the sensitivity estimates ranged from 0.14 (95% CI 0.09-0.21) (Imai, 2020) to 1.00 (95% CI 0.77-1.00) (Qiang Wang, 2020) and specificity estimates ranged from 0.41 (95% CI 0.21-0.64) (Yunbao Pan, 2020) to 1.00 (95% CI 0.97-1.00) (Bin Lou, 2020) (Fig. 5a). For the IgM based LFIA tests (*n* = 16), the sensitivity estimates ranged from 0.05 (95% CI 0.01-0.18) Adams (assay 4 to 1.00) (95% CI 0.77-1.00) (Qiang Wang, 2020) and specificity estimates ranged from 0.64 (95% CI 0.41-0.83) (Yunbao Pan, 2020) to 1.00 (95% CI 0.94-1.00) (Adams assays 4 and 5) (Fig. 5b). For the IgG-IgM based LFIA tests (*n* = 24), the sensitivity estimates ranged from 0.18 (95% CI 0.08-0.34) (Cassaniti, 2020) to 1.00 (95% CI 0.77-1.00) (Qiang Wang, 2020), with most of the studies having sensitivities over 0.55 and specificity estimates ranged from 0.36 (95% CI 0.17-0.59) (Yunbao Pan, 2020) to 1.00 (95% CI 0.94-1.00) (Adams assays 2 and 3) (Fig. 5c).

**Fig. 6:**
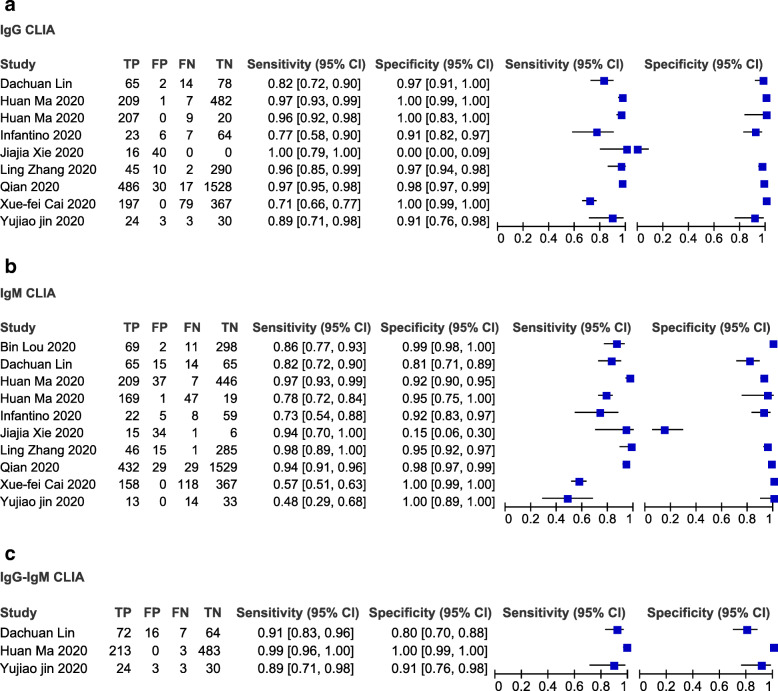
Forest plot of sensitivity, specificity and heterogeneity of serological CLIA diagnosis of COVID-19. **a** Forest plot for the IgG CLIA. **b** Forest plot for the IgM based CLIA. **c** Forest plot for the IgG-IgM based CLIA

We then considered performance of the different IgG or IgM or IgG-IgM based CLIA test using rRT-PCR-confirmed cases as the reference standard (Fig. 6a, b and c). Considering any positive result (IgM positive, IgG positive or both), CLIA serological tests achieved sensitivity ranging from 0.48 (95% CI 0.29-0.68%) (Yujiao Jin, 2020) to 1.00 (95% CI 0.79-1.00) with most studies being between 0.80 and 1. The specificity was over 0.80 in most tests except for 2 tests, one IgG based test and one IgM based test which had the lowest 0.00 (95% CI 0.00-0.009) and second lowest 0.15 (95% CI 0.06-0.30) specificities respectively.

**Fig. 7:**
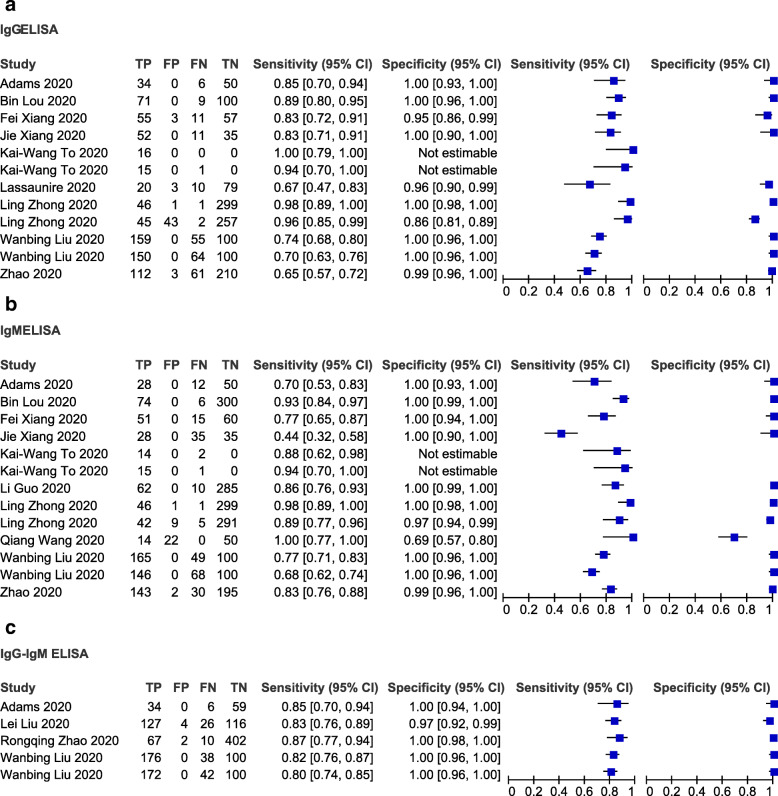
Forest plot of sensitivity, specificity and heterogeneity of serological ELISA diagnosis of COVID-19. **a** Forest plot for the IgG ELISA. **b** Forest plot for the IgM based ELISA. **c** Forest plot for the IgG-IgM based ELISA

Lastly, we evaluated the performance of the different IgG or IgM or IgG-IgM based ELISA tests using rRT-PCR-confirmed cases as the reference standard (Fig. 7a, b and c). The sensitivities and specificities were generally high, ranging from 0.80 to 1.00 and 0.95 to 1.00 in most studies. For all the IgG based ELISA tests (*n* = 10), the sensitivity estimates ranged from 0.65 (95% CI 0.57-0.72) (Zhao, 2020) to 1.00 (95% CI 0.79-1.00) (Kai-Wang To, 2020) and specificity estimates from 0.86 (95% CI 0.51-0.89) to 1.00 (95% CI 0.98-1.00) (Ling Zhong, 2020) (Fig. 7a). In the IgM based tests (*n* = 11), the sensitivity and specificity in the individual studies ranged from 0.44 (95% CI 0.32-0.58) (Jie Xiang, 2020) to 1.00 (95% CI 0.77–1.00) (Qiang Wang, 2020) and 0.69 (95% CI 0.57-0.80) (Qiang Wang, 2020) to 1.00 (95% CI 0.99–1.00) (Ling Zhong, 2020), respectively (Fig. 7b). The sensitivity across the 5 studies included in the IgG-IgM based ELISA tests ranged from 0.80 (95% CI 0.74-0.85) (Wanbing Liu, 2020) to 0.87 (95% CI 0.77-0.94) (Rongqing Zhao, 2020). On the other hand, specificity across the 5 studies ranged from 0.97 (95% CI 0.92-0.99) (Lei Liu, 2020) to 1.00 (95% CI 0.98-1.00) (Rongqing Zhao, 2020) (Fig. 7c).

**Fig. 8:**
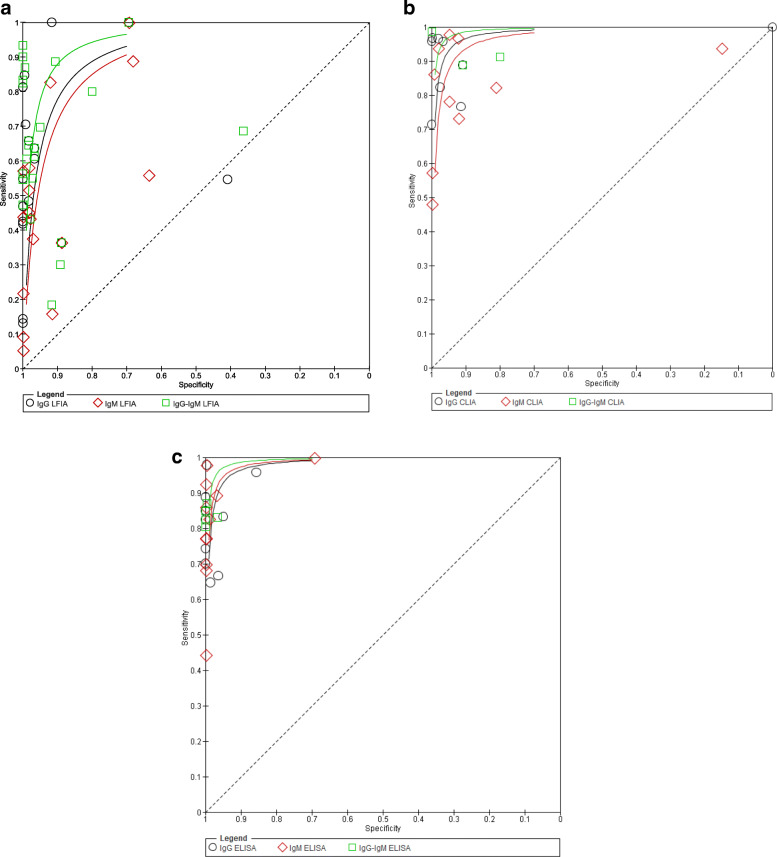
Summary ROC curves for the three antibody serological test groups. Every symbol reflects a 2 × 2 table, one for each test. One study may have contributed more than one 2 × 2 table. The curves are shown for the different test types

We also constructed the SROC curves for all the three antibody based serological tests (Fig. 8). However, we did not calculate the area under the ROC (AUROC). From the SROC, we visually assessed heterogeneity between the different tests. Diagonal line indicated useless tests and the best tests were clustered further up to the top left hand corner.

**Fig. 9:**
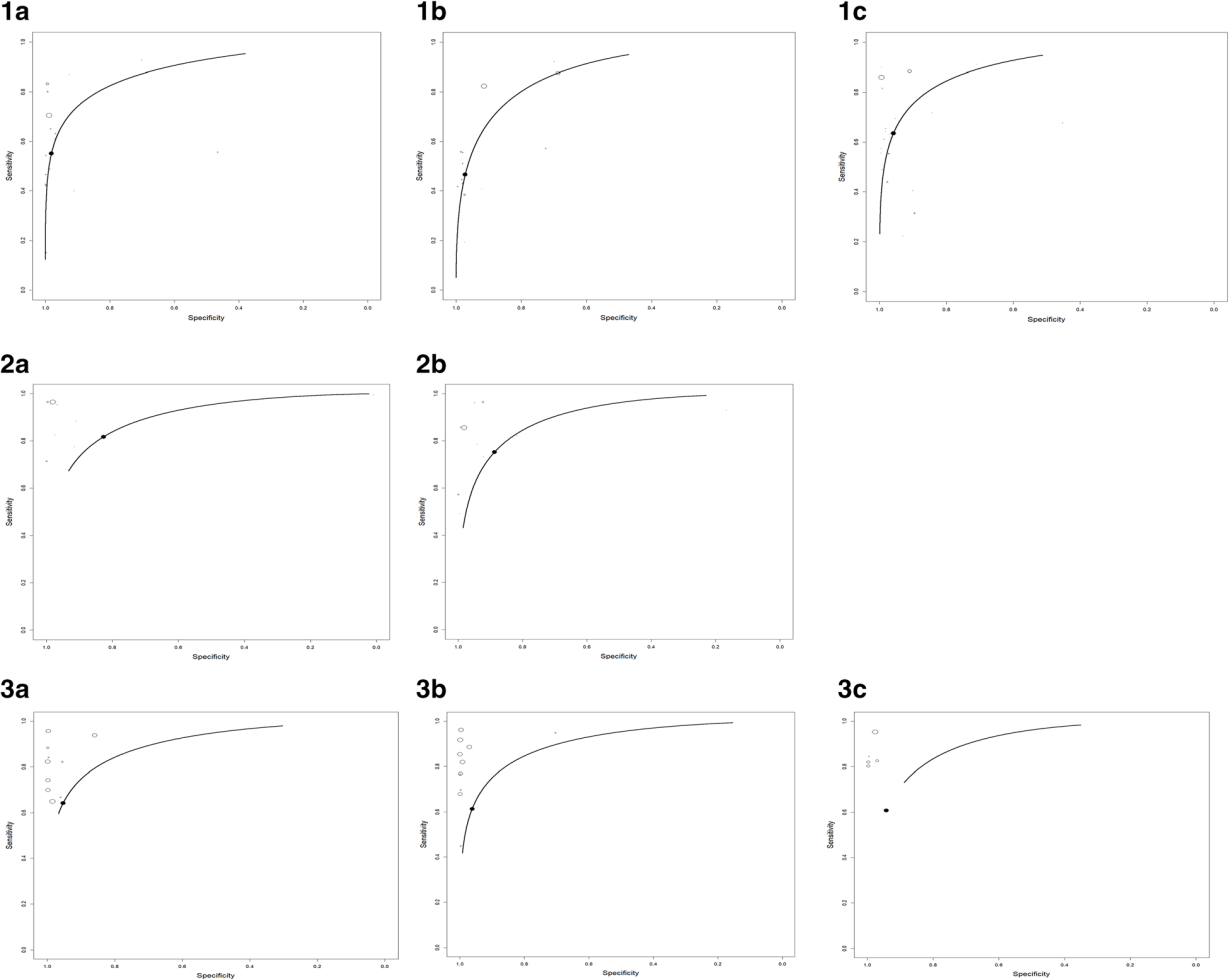
Hierarchical summary receiver operating characteristic (HSROC) curve obtained using OpenMeta-Analyst. Every circle represents the sensitivity and specificity estimates of individual studies in the meta-analysis, and the size of the circle reflects the sample size. The black dots indicate summary points of sensitivity and specificity; HSROC curve is the line passing through summary point. The curve is the regression line that summarises the overall diagnostic accuracy. **a** HSROC for IgG serological tests. **b** HSROC for IgM serological tests. **c** HSROC IgG-IgM serological tests. 1, LFIA HSROC; 2, CLIA HSROC and 3, ELISA HSROC

The bivariate model and the hierarchical summary receiver operating characteristic curve (HSROC) model were performed to evaluate the diagnostic accuracy of the serological tests. The outputs of the meta-analysis (bivariate and HSROC parameter estimates, as well as the summary values of sensitivity and specificity) are presented in Table 2 and Fig. 9. The pooled sensitivity for the IgG, IgM and IgG-IgM based LFIA tests were 0.5856, 0.4637 and 0.6886 respectively compared to rRT-PCR. The pooled sensitivity for the IgG and IgM based CLIA tests were 0.9311 and 0.8516 respectively compared to rRT-PCR. The pooled sensitivity for the IgG, IgM and IgG-IgM based ELISA tests were 0.8292, 0.0.8388 and 0.8531 respectively compared to rRT-PCR. All the tests had high specificities ranging from 0.9693 to 0.9991 compared to rRT-PCR. The estimated SROC curves for bivariate models are not presented.

**Table 2:** Summary estimates of test accuracy

Test type	Antibody type	Number of studies/tests	Sensitivity (95% CI)	Specificity (95% CI)	Correlation
LFIA	IgG	17	58.56 (43.97-71.79)	98.96 (95.61-99.76)	−0.4454
CLIA	IgG	9	93.11 (93.09-93.12)	97.57 (97.57-97.58	−0.511
ELISA	IgG	10	82.92 (74.16-89.15)	99.48 (96.75-99.92)	−0.1709
LFIA	IgM	16	46.37 (30.16-0.6339)	97.34 (92.75-99.05)	−0.7925
CLIA	IgM	10	85.16 (73.56-0.9221)	96.93 (85.5-99.41)	−0.7074
ELISA	IgM	11	83.88 (0.7307-0.909)	99.91 (97.78-100)	−0.7247
LFIA	IgG-IgM	24	68.86 (58.78-77.42)	97.57 (94.66-98.92)	0.1011
CLIA	IgG-IgM	3			
ELISA	IgG-IgM	5	85.31 (78.51-90.23)	99.01 (92.87-99.87)	−0.6771

HSROCs were also used to visually access the overall performance of the diagnostic tests, to access the overall diagnostic accuracy of the tests and to compare the diagnostic accuracy of the different tests used for diagnosing COVID-19 in the review (Fig. 9). The overall diagnostic test accuracy was measured by the proximity of the curve to the top left corner which represents high sensitivity and specificity. The closer the curve was to the upper left hand corner, the better the diagnostic accuracy [55]. From Fig. 9, it can be observed that ELISA and CLIA have better diagnostic accuracy compared to LFIA and IgG-IgM based ELISA tests have the best overall diagnostic test accuracy. Of importance, it is noteworthy that in the study the evidence base was too weak to definitively state that one class of test was more accurate than the other class of tests.

We identified one study (Burbelo, 2020) reporting total antibody (Ab) based luciferase immunoprecipitation assay system (LIPS) using N and S antigens with sensitivities and specificities of 0.91 (95% CI 0.77-0.99) and 1.00 (0.80-1.00) and 1.00 (0.92-1.00) and 1.00 (0.92-1.00) respectively. We also identified studies reporting other Ab based serological assays and IgA based serological assays but results are not reported in this review.

### Heterogeneity investigations

Generally high overall *I*^^2^ values above 85%, which indicate high heterogeneities, were observed for both the sensitivities and specificities when we performed antigen subgroup meta-analysis with the exception of IgG-IgM based ELISA. IgG-IgM based ELISA had an overall sensitivity *I*^^2^ value of 52. 12% which is considered moderate heterogeneity and overall specificity *I*^^2^ value of 0% which is considered to be low heterogeneity. However, it should be noted that only 5 studies were included for this subgroup meta-analysis. Overall *I*^^2^ values for sensitivities and specificities heterogeneities for the antigen type subgroup meta-analysis are shown in Table 3. We did not investigate heterogeneity for LFIA because most studies included in the review did not specify the type of antigen they used in their serological tests.

**Table 3:** Overall antibody type subgroup meta-analysis heterogeneity

Test type	Antibody type	Heterogeneity (*I*^^2^ )	
		Sensitivity	Specificity
LFIA	IgG		
	IgM		
	IgG-IgM		
CLIA	IgG	93.56%	86.5%
	IgM	93.42%	95.17%
	IgG-IgM		
ELISA	IgG	78.07%	84.97%
	IgM	85.47%	90.08%
	IgG-IgM	52.12%	0%

Detailed results of heterogeneity for the different antigen type sensitivities and specificities for each test type and antibody type combination are presented in Additional file 2.

### Test sensitivity by time since onset of symptoms

**Fig. 10:**
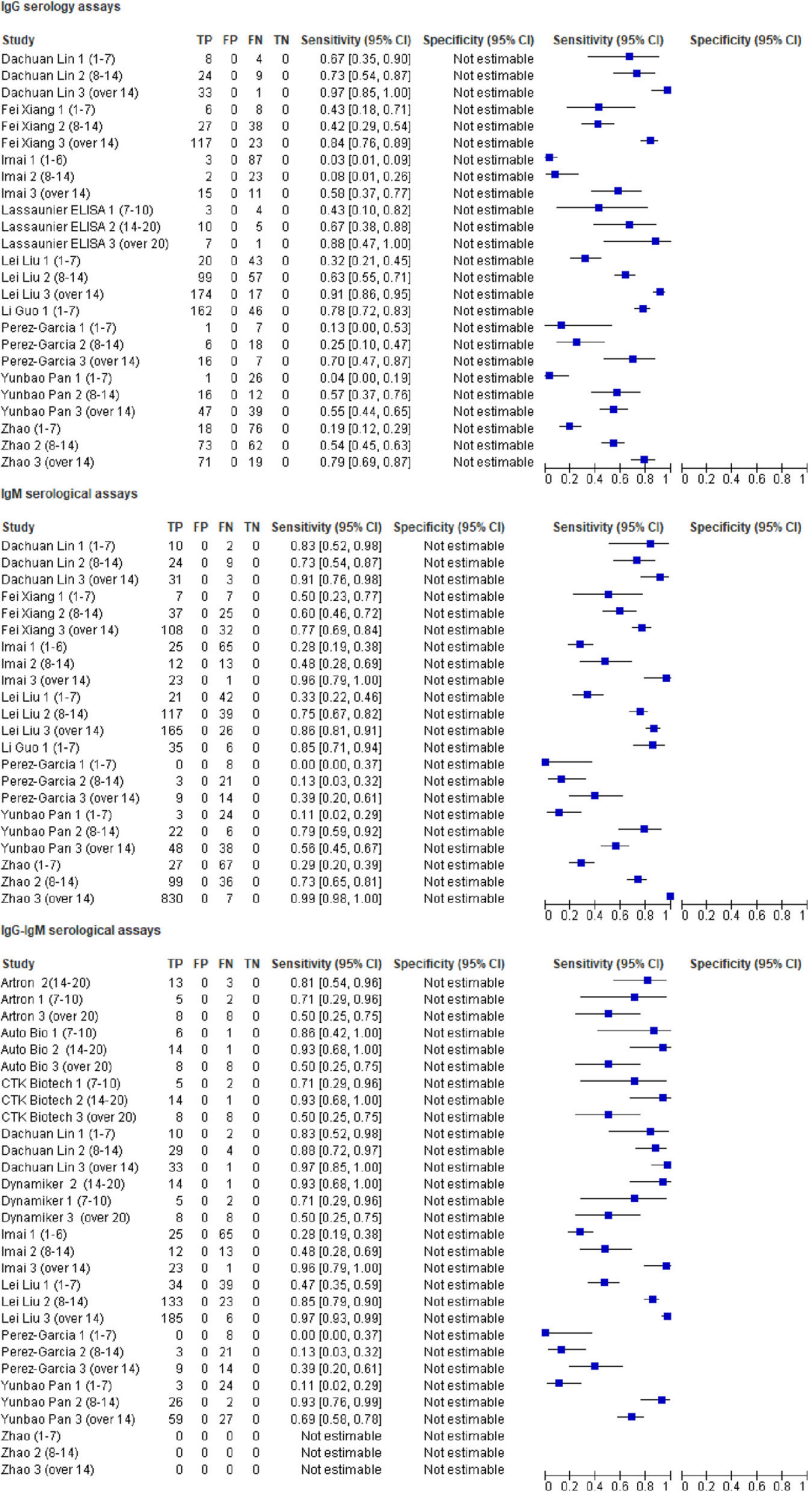
Forest plot of studies evaluating tests for detection of IgG, IgM and IgG-IgM according to days since COVID-19 symptom onset to specimen collection. In brackets () are the number of days since symptom onset to specimen collection. Artron, Auto Bio CTK Biotech CTK Biotech are test names all reported in a study by Lassaunire et al

Figure 10 shows forest plots for antibody positive rates for IgG (25 tests), IgM (22 tests) and IgG-IgM (30 tests), stratified by days since initial symptom onset to specimen collection. The sensitivity of the serological tests generally increased with increased time from symptoms onset. Regardless of test method (ELISA or CLIA or LFIA), the sensitivities for IgG and IgM based tests were generally low in the first week (1-7 days) of symptom onset followed by the second (7-14 days) and the sensitivities were generally highest in the third week or later (>14 days) for each test. Data on specificity stratified by specimen collection since symptom onset was not available for all the studies.

## Discussion

COVID-19 is a major healthcare challenge globally. One key aspect of limiting SARS-CoV-2 spread is to ensure early and accurate diagnosis of the viral infection. In this study, we performed a meta-analysis to evaluate the diagnostic accuracy of IgG and IgM based serological assays offered to detect antigens against rRT-PCR positive SARS-CoV-2 patients. The meta-analysis showed that all serological assays yielded high specificities ranging from 0.9693 (95% CI 0.855-0.9941) to 0.9991 (95% CI 0.9778-1) in comparison to rRT-PCR. Meta-analysis by Wang et al., Bastos et al. and Deeks et al. observed similar pooled specificities ranging from 0.95 (95% CI 0.91–0.98) to 99.6 (97.3 to 99.9) [56–58]. The sensitivities of all serological assays varied widely across studies. COVID-19 serological assays rely on antibodies binding to SARS-CoV-2 nucleocapsid protein, spike protein or spike protein fragments (i.e. receptor binding domain). Some tests or test devices even use a combination of the N and S proteins and protein fragments. This may have resulted in the inconsistencies observed in the different serological assays [59]. Overall IgG-IgM based ELISA had superior diagnostic accuracy with sensitivity and specificity of 0.8531 (95% CI 0.7851-0.9023) and 0.9991 (95% CI 0.9778-1). IgG based CLIA had the highest sensitivity 0.9311 (95% CI 0.9309-0.9312). The pooled sensitivity results are in agreement with other meta-analysis that showed POC LFIA had lower sensitivities than CLIA and ELISA within each antibody class [56–58]. However, it must be noted that a meta-analysis by Deeks et al. found that for IgG and IgG-IgM, concentration gradient immunoassay POC LFIA had higher pooled sensitivity than ELISA [56].

Similar to other meta-analysis, IgM based serological assays had the lowest sensitivities compared IgG based serological tests in each respective test method [56–58]. Low antibody concentrations and especially timing of the IgM based serological assays could potentially explain the low ability of the IgM based tests to identify people infected with SARS-CoV-2. Notably, immediately after a person is infected, antibodies may not have been developed yet or too late IgM antibodies may have decreased or disappeared [60].

Generally combined IgG-IgM based serological assays seem to be a better choice in terms of sensitivity than measuring either antibody type alone, though in this review we did not estimate the pooled sensitivity of the IgG-IgM based CLIA due to the limited number of studies. Subgroup meta-analysis using Open Meta-Analyst showed that IgM based serological assays that use the S antigen are more sensitive than IgM based serological assays that used the N antigen-based tests probably due to higher sensitivity and earlier immune response to the S antigen [32]. However, subgroup meta-analysis showed that IgG based serological assays that use N antigen are more sensitive than IgG based serological assays that use S antigen.

The sensitivity and specificity estimates from this review were used to indicate the consequences of testing when the test is being used in clinical practice. Prevalence estimates were calculated using metaDTA [61] to predict how many patients in practice you would expect to have true positive, false positive, true negative and false negative results for a given prevalence based on the meta-analysis results giving the results some clinical context. Using IgG-IgM based ELISA test which had the best overall diagnostic accuracy as an example, one can see that with 1000 patients and a COVID-19 prevalence of 50%, we would expect 432 (95% CI 394-486) patients to test positive for SARS-CoV-2, of which 427 (95% CI 393-451) will be true positives (are diseased and test positive) and 5 (95% CI 1-35) will be false positives (are not diseased but test positive). We also noted 568 (95% CI 514-606) patients test negative for COVID-19, of which 495 (95% CI 465-499) were true negatives (are not diseased and test negative) and 73 (95% CI 49-107) were false negatives (are diseased but test negative).

A prevalence of 1% and 1000 patients would mean that 19 (95% CI 9-79) patients test positive for SARS-CoV-2, 9 (95% CI 8-9) of these will be true positives and 10 (95% CI 1-70) false positives and 981 (95% CI 921-991) patients will test negative for SARS-COV-2, 980 (95% CI 920-989) of these will be true negatives and 1 (95% CI 1-2) patient will be false negative. This means that the false positive will be treated for COVID-19 whilst they have another disease, thus delaying their final diagnosis and subsequent treatment. In the false-negatives, COVID-19 diagnosis will be missed or delayed and the patients will not be quarantined and they will thus spread the SARS-CoV-2 to other patients in the hospital/clinic. Nonetheless, despite calculations like these providing insight in the consequences of testing, they should be taken with caution.

Using the inferences for LIFA using the IgG-IgM based test [sensitivity 0.6886 (0.5878-0.7742) and specificity 0.9757 (0.9466-0.9892)]. We see that for 1000 patients and a COVID-19 prevalence of 50%, we would expect 356 (95% CI 299-414) patients to test positive for SARS-CoV-2, 324 (95% CI 294-387) of which will be true positives and 12 (95% CI 5-27) will be false positives. We would also expect 644 (95% CI 586-701) patients to test negative for the COVID-19, 488 (95% CI 473-495) of which will be true negatives and 156 (95% CI 113-206) are false negatives. A prevalence of 1% and 1000 patients would mean 31 (95% CI 16-62) patients test positive for SARS-CoV-2, 7 (95% CI 6-8) of these will be true positives and 24 (95% CI 10-54) false positives and 969 (95% CI 938-984) patients to test negative for COVID, 966 (95% CI 936-980) of these will be true negatives and 3 (95% CI 2-4) are false negatives. Compared to IgG-IgM based ELISA, the IgG-IgM based LFIA has higher rates of false positives and false negatives.

### Limitation

Serological tests for SARS-CoV-2 have accuracy issues that warrant attention. They measure specific antibody responses which may take some weeks to develop after disease onset reducing the sensitivity of the assay. If blood samples were collected during the early stage of the infection, they may produce false negative results. They do not directly detect the presence of the virus. Further, antibodies may be present when SARS-CoV-2 is no longer present giving false positive case diagnosis. Moreover, since the identity of the N protein of SARS-CoV-2 and SARS-CoV reached up to 91.2%, there is probability of a cross reaction between the N protein of SARS-CoV-2 and antibodies against other human coronaviruses. Other molecules including interferon, rheumatoid factor and non-specific IgM may cause false positive results [42].

Most studies included in the meta-analysis were case-control studies. These may be easy to perform in a laboratory setting than cross-sectional designs, but their results are less representative for clinical practice. The performance of diagnostic tests very much depends on the population in which the test is being used. Future studies should therefore be prospective cross-sectional studies including a consecutive sample of presenting patients.

Index tests need to be evaluated to determine their sensitivity and specificity, ideally by comparison with a standard confirmatory test. An important limitation with the rRT-PCR, the standard confirmatory test for COVID-19 is the risk of false-negative results [62]. Two reviews of the accuracy of rRT-PCR COVID-19 tests reported false negative rates of between 2% and 29%, based on negative rRT-PCR tests which were positive on repeat rRT-PCR testing [63, 64]. False negative results of rRT-PCR tests can lead decreased specificity of the serological tests (index tests). The rRT-PCR negative results picked up as positive tests by the serological tests will be treated as false positives thereby lowering the specificity of the serological tests. In order to reduce false-negative results, Bastos et al. recommended that the standard confirmatory test should consist of RT-PCR performed on at least two consecutive specimens and when possible it must include viral cultures [57].

## Conclusion

Given the poor performance of the current LFIA devices, we recommend more research to develop highly sensitivity and specific POC LFIA that are adequate for most individual patient applications and attractive for large seroprevalence studies. The use of CLIA and ELISA for diagnosis has high sensitivity and is comparable to using rRT-PCR. They may be calibrated to be specific for detecting and quantifying SARSCoV-2 IgM and IgG. More serological data should be collected to elucidate the clinical and epidemiological utility of IgG and IgM serological measurements to detect symptomatic and asymptomatic cases of COVID-19.

### Supplementary Information

**Additional file 1.** PRISMA-DTA Checklist

**Additional file 2.** Antigen type subgroup-meta analysis results

## Data Availability

The raw data (data extraction results) will be provided for sharing after reasonable request.

## Appendix 1

### Search strategy in PubMed

**Table:**

Search	Query	Items found
#20	Search (((((COVID-19[Title/Abstract]) OR SARS-CoV-2[Title/Abstract]) OR 2019-nCoV[Title/Abstract]) OR Wuhan Coronavirus[Title/Abstract])) AND (((((((((((((Serologic test) OR Serologic method) OR Serological test) OR Serological method) OR Serodiagnosis) OR Immunodiagnosis) OR Immunological test) OR Immunological method) OR Antibody detection) OR Antigen detection) OR IgM) OR IgG) OR Immunochromatography)	78
#19	Search ((((((((((((Serologic test) OR Serologic method) OR Serological test) OR Serological method) OR Serodiagnosis) OR Immunodiagnosis) OR Immunological test) OR Immunological method) OR Antibody detection) OR Antigen detection) OR IgM) OR IgG) OR Immunochromatography	822733
#18	Search Immunochromatography	69624
#17	Search IgG	143941
#16	Search IgM	66041
#15	Search Antigen detection	82456
#14	Search Antibody detection	99123
#13	Search Immunological method	39637
#12	Search Immunological test	456120
#11	Search Immunodiagnosis	450622
#10	Search Serodiagnosis	184574
#9	Search Serological method	24322
#8	Search Serological test	191599
#7	Search Serologic method	16939
#6	Search Serologic test	184758
#5	Search (((COVID-19[Title/Abstract]) OR SARS-CoV-2[Title/Abstract]) OR 2019-nCoV[Title/Abstract]) OR Wuhan Coronavirus[Title/Abstract]	6734
#4	Search Wuhan Coronavirus[Title/Abstract]	14
#3	Search 2019-nCoV[Title/Abstract]	547
#2	Search SARS-CoV-2[Title/Abstract]	1898
#1	Search COVID-19[Title/Abstract]	5985

## Appendix 2

### Rating of QUADAS-2 items

#### 1. Patient selection

1a. Risk of bias, signalling questions

- Was a case-control design avoided?
  - ▪ Case-control designs, especially if they include healthy controls, carry a high risk of bias. Therefore, all case-control studies are automatically rated to be of high risk of bias in the overall judgement.
- Was a consecutive or random sample of patients enrolled?
- Did the study avoid inappropriate exclusions?
- Overall judgement:
  - ▪ Case-control studies were always rated as having a high risk of bias.
  - ▪ Cross-sectional studies: only low risk of bias if the other two signalling questions are answered with ‘yes’. If one of the questions was answered ‘no’, then high risk of bias. Otherwise ‘unclear’.

1b. Concerns regarding applicability: this concerns the extent to which the patients (both cases and controls) that were included in a study are representative for the patients which will receive these serology tests.

- Is there concern that the included patients do not match the review question?
  - ▪ All case-control studies are by default rated ‘high concern’. All cross-sectional studies are by default ‘low concern’, except when the used case definition was not very clear.

#### 2. Index test

2a. Risk of bias, signalling questions

- Were the index test results interpreted without knowledge of the results of the reference standard?
- If a threshold was used, was it pre-specified? By selecting the cut-off value with the highest sensitivity and/or specificity, researchers artificially optimise the accuracy of their tests, which may lead to an overestimation of sensitivity and specificity. POC LFIA threshold were pre-specified and the signal was the development of a line or colour.
- Overall judgement:
  - ▪ If a study was not reporting results from automated assays and it did not explicitly mention blinding then tests were automatically rated as high risk.
  - ▪ If there was blinding and the second question was answered with ‘yes’, overall judgement was ‘low’.

2b. Concerns regarding applicability: this concerns the extent to which the index test evaluated is representative of the tests that will be used in practice.

- Are there concerns that the index test, its application or interpretation deviate from the review question?
  - ▪ All in-house tests were automatically rated as ‘high concern’.
  - ▪ If serum or plasma samples not blood were used for POC LFIA then tests were automatically rated high concern

Risk of bias and concerns regarding applicability were assessed for each test separately.

#### 3. Reference standard

3a. Risk of bias, signalling questions

- Is the reference standard likely to correctly classify the target condition?
  - ▪ Assumption: This will likely be the case for case-control studies that use the ‘correct’ case definitions
  - ▪ This is also likely for cross-sectional studies which use the ‘correct’ case definitions.
- Were the reference standard results interpreted without knowledge of the results of the index test?
  - ▪ Assumption: This will likely be the case for most case-control studies, but only if serology was not part of the case definition.
  - ▪ For cross-sectional studies, this should be explicitly stated.
- Overall judgement risk of bias:
  - ▪ Case-control studies with clear case definitions were rated as having a ‘low’ risk of bias.
  - ▪ case-control studies with unclear/wrong case definitions rated as ‘unclear’ Or ‘high risk’ of bias respectively
  - ▪ Cross-sectional studies with a clear case definition and the second question answered with ‘yes’: low risk of bias.
  - ▪ Otherwise ‘unclear’.

3b. Concerns regarding applicability: Are there concerns that the target condition as defined by the reference standard does not match the review question?

- ▪ If serology is included in the case definition, there is an incorporation bias and thus a high risk of bias.
- ▪ If a case-control study uses clear criteria and does not include serology in these criteria: ‘low’ concern of bias.

#### 4. Risk of bias regarding flow and timing, signalling questions

- Was there an appropriate interval between index test(s) and reference standard?
  - ▪ We expected that studies with a cross-sectional design conducted most tests on a date sufficiently close to the final diagnosis. If we had reason to suspect that the patient status changed between the time of testing and the time of diagnosis, we rated this as ‘no’.
  - ▪ For case-control studies, this was always rated as ‘no’, because serology was always determined after the case definitions were defined, sometimes with a long delay.
- Did patients receive the same reference standard?
- Were all patients included in the analysis?
  - ▪ This was rated ‘no’ for all case-control studies.
- Overall judgement:
  - ▪ Case-control studies were always rated as having a high risk of bias.
  - ▪ For cross-sectional studies, we perceived a low risk of bias if all three questions were answered with ‘yes’; a high risk of bias was perceived if at least one of them was answered ‘no’. All other possibilities were rated as ‘unclear’ [65].

## Abbreviations

COVID-19: Coronavirus disease 2019
SARS: Severe acute respiratory syndrome
SARS-CoV-2: Severe acute respiratory syndrome coronavirus 2
STARD: Standards for the reporting of diagnostic accuracy studies
QUADAS-2: Quality Assessment of Diagnostic Accuracy Studies-2
rRT-PCR: Real-time reverse-transcriptase polymerase chain reaction
PRISMA-DTA: Preferred Reporting Items for Systematic Reviews and Meta-Analyses of Diagnostic test accuracy
DTA: Diagnostic test accuracy
RT-qPCR: Quantitative real-time polymerase chain reaction
POC: Point-of-care
LFIA: Lateral flow immunoassays
CLIA: Chemiluminescence enzyme immunoassay
FIA: Fluorescence
EISA: Enzyme-linked immunoassay
LIPS: Luciferase immunoprecipitation assay systems
S: SARS-CoV-2 spike protein
N: SARS-CoV-2 nucleocapsid protein
E: SARS-CoV-2 envelope protein
HSROC: Hierarchical summary receiver operating characteristic
TP: True positive
FN: False negative
FP: False positive
TN: True negative
