# Supplementary material for: A systematic and meta-analysis review on the diagnostic accuracy of antibodies in the serological diagnosis of COVID-19
Source: Syst Rev. 2021 May 26;10:155. doi: 10.1186/s13643-021-01689-3 (PMC8152206; doi:10.1186/s13643-021-01689-3)
Supplement: Supplementary file 2 — Additional file 2. Antigen type subgroup-meta analysis results [file 13643_2021_1689_MOESM2_ESM.pdf]

Additional table 1: Chemiluminescene Immunoassays SubgroupMeta-analysis

a: IgG based CLIA

Diagnostic Random-Effects Model

Metric: Sensitivity
Model Results

Subgroups	Studies	Estimate	Lower bound	Upper bound	Std. error	p-Val	z-Val
Subgroup S	2	0.894	0.423	0.990	1.243	0.087	1.711
Subgroup N	2	0.909	0.687	0.979	0.774	0.003	2.973
Subgroup S-N	3	0.904	0.681	0.977	0.758	0.003	2.959
Subgroup Not stated	1	0.957	0.845	0.989	0.723	NA	NA
Subgroup E-N	1	0.971	0.664	0.998	1.435	NA	NA
Overall	9	0.915	0.818	0.963	0.446	< 0.001	5.327

Heterogeneity

Studies	Q (df)	Het. p-Val	I^2
Subgroup S	37.405 (1)	< 0.001	97.33 %
Subgroup N	12.178 (1)	< 0.001	91.79 %
Subgroup S-N	20.236 (2)	< 0.001	90.12 %
Subgroup Not stated	NA	NA	NA
Subgroup E-N	NA	NA	NA
Overall	124.266 (8)	< 0.001	93.56 %

Diagnostic Random-Effects Model

Metric: Specificity
Model Results

Subgroups	Studies	Estimate	Lower bound	Upper bound	Std. error	p-Val	z-Val
Subgroup S	2	0.998	0.991	1.000	0.817	< 0.001	7.732
Subgroup N	2	0.975	0.918	0.993	0.640	< 0.001	5.736
Subgroup S-N	3	0.950	0.845	0.985	0.637	< 0.001	4.619
Subgroup Not stated	1	0.967	0.939	0.982	0.322	NA	NA
Subgroup E-N	1	0.012	0.001	0.167	1.423	NA	NA
Overall	9	0.961	0.901	0.985	0.501	< 0.001	6.376

Heterogeneity

Studies	Q (df)	Het. p-Val	I^2
Subgroup S	0.059 (1)	0.808	0 %
Subgroup N	0.001 (1)	0.975	0 %
Subgroup S-N	16.134 (2)	< 0.001	87.6 %
Subgroup Not stated	NA	NA	NA
Subgroup E-N	NA	NA	NA
Overall	59.249 (8)	< 0.001	86.5 %

b: IgM CLIA

Diagnostic Random-Effects Model

Metric: Sensitivity
Model Results

Subgroups	Studies	Estimate	Lower bound	Upper bound	Std. error	p-Val	z-Val
Subgroup S	3	0.860	0.494	0.975	0.938	0.053	1.935
Subgroup N	2	0.793	0.742	0.835	0.144	< 0.001	9.317
Subgroup S-N	3	0.721	0.442	0.894	0.603	0.115	1.576
Subgroup Not stated	1	0.979	0.864	0.997	1.011	NA	NA
Subgroup E-N	1	0.938	0.665	0.991	1.033	NA	NA

Overall	10	0.829	0.724	0.900	0.314	< 0.001	5.024

Heterogeneity

Studies	Q (df)	Het. p-Val	I^2
Subgroup S	72.825 (2)	< 0.001	97.25 %
Subgroup N	0.573 (1)	0.449	0 %
Subgroup S-N	24.053 (2)	< 0.001	91.69 %
Subgroup Not stated	NA	NA	NA
Subgroup E-N	NA	NA	NA
Overall	136.827 (9)	< 0.001	93.42 %

Diagnostic Random-Effects Model

Metric: Specificity
Model Results

Subgroups	Studies	Estimate	Lower bound	Upper bound	Std. error	p-Val	z-Val
Subgroup S	3	0.988	0.885	0.999	1.220	< 0.001	3.629
Subgroup N	2	0.867	0.641	0.960	0.662	0.005	2.835
Subgroup S-N	3	0.968	0.899	0.990	0.627	< 0.001	5.454
Subgroup Not stated	1	0.950	0.919	0.970	0.265	NA	NA
Subgroup E-N	1	0.150	0.069	0.296	0.443	NA	NA
Overall	10	0.942	0.854	0.978	0.519	< 0.001	5.363

Heterogeneity

Studies	Q (df)	Het. p-Val	I^2
Subgroup S	19.643 (2)	< 0.001	89.82 %
Subgroup N	1.925 (1)	0.165	48.06 %
Subgroup S-N	8.987 (2)	0.011	77.75 %
Subgroup Not stated	NA	NA	NA
Subgroup E-N	NA	NA	NA

Overall	186.307 (9)	< 0.001	95.17 %

Additional table 2: ELISA Subgroup Meta-analysis

a: IgG ELISA

Diagnostic Random-Effects Model

Metric: Sensitivity
Model Results

Subgroups	Studies	Estimate	Lower bound	Upper bound	Std. error	p-Val	z-Val
Subgroup S	5	0.761	0.656	0.842	0.262	< 0.001	4.424
Subgroup N	4	0.851	0.709	0.930	0.434	< 0.001	4.009
Subgroup Not specified	1	0.820	0.707	0.896	0.326	NA	NA
Overall	10	0.798	0.730	0.852	0.192	< 0.001	7.138

Heterogeneity

Studies	Q (df)	Het. p-Val	I^2
Subgroup S	17.238 (4)	0.002	76.79 %
Subgroup N	19.415 (3)	< 0.001	84.55 %
Subgroup Not specified	NA	NA	NA
Overall	41.038 (9)	< 0.001	78.07 %

Diagnostic Random-Effects Model

Metric: Specificity
Model Results

Subgroups	Studies	Estimate	Lower bound	Upper bound	Std. error	p-Val	z-Val
Subgroup S	5	0.972	0.889	0.993	0.739	< 0.001	4.776
Subgroup N	4	0.990	0.950	0.998	0.840	< 0.001	5.453
Subgroup Not specified	1	0.998	0.973	1.000	1.415	NA	NA
Overall	10	0.985	0.954	0.995	0.589	< 0.001	7.119

Heterogeneity

Studies	Q (df)	Het. p-Val	I^2
Subgroup S	28.638 (4)	< 0.001	86.03 %
Subgroup N	7.748 (3)	0.052	61.28 %
Subgroup Not specified	NA	NA	NA
Overall	59.869 (9)	< 0.001	84.97 %

b: IgM ELISA

Diagnostic Random-Effects Model

Metric: Sensitivity
Model Results

Subgroups	Studies	Estimate	Lower bound	Upper bound	Std. error	p-Val	z-Val
Subgroup S	5	0.824	0.748	0.881	0.233	< 0.001	6.621
Subgroup N	4	0.814	0.677	0.901	0.376	< 0.001	3.920
Subgroup Not specified	2	0.785	0.102	0.992	1.772	0.465	0.731
Overall	11	0.800	0.718	0.863	0.232	< 0.001	5.985

Heterogeneity

Studies	Q (df)	Het. p-Val	I^2
Subgroup S	13.756 (4)	0.008	70.92 %
Subgroup N	16.503 (3)	< 0.001	81.82 %
Subgroup Not specified	6.034 (1)	0.014	83.43 %
Overall	68.840 (10)	< 0.001	85.47 %

Diagnostic Random-Effects Model

Metric: Specificity
Model Results

Subgroups	Studies	Estimate	Lower bound	Upper bound	Std. error	p-Val	z-Val
Subgroup S	5	0.988	0.967	0.995	0.514	< 0.001	8.524

Subgroup N	4	0.996	0.987	0.999	0.634	< 0.001	8.789
Subgroup Not specified	2	0.905	0.252	0.996	1.704	0.186	1.322
Overall	11	0.989	0.957	0.997	0.727	< 0.001	6.229

Heterogeneity

Studies	Q (df)	Het. p-Val	I^2
Subgroup S	6.833 (4)	0.145	41.46 %
Subgroup N	0.651 (3)	0.885	0 %
Subgroup Not specified	5.702 (1)	0.017	82.46 %
Overall	100.762 (10)	< 0.001	90.08 %

c: IgG-IgM ELISA

Diagnostic Random-Effects Model

Metric: Sensitivity
Model Results

Subgroups	Studies	Estimate	Lower bound	Upper bound	Std. error	p-Val	z-Val
Subgroup S	3	0.881	0.754	0.947	0.449	< 0.001	4.459
Subgroup N	2	0.813	0.770	0.850	0.134	< 0.001	10.989
Overall	5	0.834	0.784	0.875	0.168	< 0.001	9.634

Heterogeneity

Studies	Q (df)	Het. p-Val	I^2
Subgroup S	7.242 (2)	0.027	72.38 %
Subgroup N	0.453 (1)	0.501	0 %
Overall	8.354 (4)	0.079	52.12 %

Diagnostic Random-Effects Model

Metric: Specificity
Model Results

Subgroups	Studies	Estimate	Lower bound	Upper bound	Std. error	p-Val	z-Val

Subgroup S	3	0.978	0.961	0.988	0.305	< 0.001	12.494
Subgroup N	2	0.980	0.901	0.996	0.856	< 0.001	4.537
Overall	5	0.977	0.962	0.986	0.257	< 0.001	14.565

Heterogeneity

Studies	Q (df)	Het. p-Val	I^2
Subgroup S	1.562 (2)	0.458	0 %
Subgroup N	1.652 (1)	0.199	39.47 %
Overall	3.368 (4)	0.498	0 %
